# Molecular epidemiology and diagnostics of KRAS mutations in human cancer

**DOI:** 10.1007/s10555-020-09915-5

**Published:** 2020-07-29

**Authors:** Jozsef Timar, Karl Kashofer

**Affiliations:** 1 2nd Department of Pathology, Semmelweis University, Budapest, Hungary; 2 Diagnostic and Research Institute of Pathology, Medical University of Graz, Auenbruggerpl. 2, 8036 Graz, Austria

**Keywords:** KRAS mutation, Human cancer, Epidemiology, Tumor progression, Diagnostics

## Abstract

RAS mutation is the most frequent oncogenic alteration in human cancers. KRAS is the most frequently mutated followed by NRAS. The emblematic KRAS mutant cancers are pancreatic, colorectal, lung adenocarcinomas and urogenital cancers. KRAS mutation frequencies are relatively stable worldwide in various cancer types with the one exception of lung adenocarcinoma. The frequencies of KRAS variant alleles appears cancer type specific, reflecting the various carcinogenic processes. In addition to point mutation KRAS, allelic imbalances are also frequent in human cancers leading to the predominance of a mutant allele. KRAS mutant cancers are characterized by typical, cancer-type-specific co-occurring mutations and distinct gene expression signatures. The heterogeneity of KRAS mutant primary cancers is significant, affecting the variant allele frequency, which could lead to unpredictable branching development in metastases. Selection of minute mutant subclones in the primary tumors or metastases during target therapies can also occur frequently in lung or colorectal cancers leading to acquired resistance. Ultrahigh sensitivity techniques are now routinely available for diagnostic purposes, but the proper determination of mutant allele frequency of KRAS in the primary or metastatic tissues may have larger clinical significance.

## Introduction

### Molecular epidemiology

The RAS proteins are members of the small GTPase protein family and serve as a binary switch in the signal transduction of most growth factor receptors including epidermal growth factor receptors (EGFRs), tyrosine kinase receptor for HGF (MET), or tyrosine kinase receptor for SCF (KIT). This family has three members, KRAS, HRAS and NRAS, the genes of which are located on different chromosomes. KRAS gene was identified as a homolog of the Kirsten rat **s**arcoma virus responsible for malignant transformation of rodent cells [1–3]. Human KRAS gene is located on chromosome 12p12.1 encoded by 6 exons. KRAS protein is evenly expressed by most tissues but overexpressed only by a few: skeletal muscle and myocardium, uterus, adrenal cortex, and certain bone marrow stem cells, otherwise rarely involved in KRAS-related carcinogenesis www.proteinatlas.org**.** HRAS gene is located on chromosome 11p15.5 and the protein is expressed by almost all tissues at low levels and overexpressed only by uterine and muscle tissue, bronchial epithelium, and Langerhans islets of the pancreas [4]. The third member of this family is the N-RAS, the gene of which is located on chromosome 1p13.2. NRAS expression is high in the GI-tract, in bone marrow, as well as in brain and endocrine tissues. www.proteinatlas.org.

**Fig 1.**
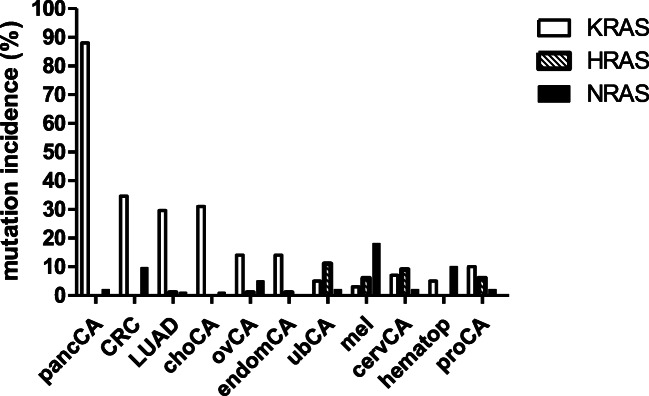
Mutation incidence of the RAS family genes in human cancers (5–7). Data are expressed in %. *cervCA* cervical cancer, *choCA* cholangial cancer, *CRC* colorectal cancer, *endomCA* endometrial cancer, *hematop* hematopoietic malignancies, *LUAD* lung adenocarcinoma, *mel* malignant melanoma, *ovCA* ovarian cancer, *pancCA* pancreatic cancer, *proCA* prostate cancer

RAS oncogenes are the most frequently mutated genes in human cancer, but RAS-driven tumors are not associated with those tissues where these genes are normally expressed. KRAS is the most frequently mutated oncogene in humans: more than 80% of pancreatic cancers and more than 30% of colorectal and cholangial cancers and lung adenocarcinomas harbor activating mutations of KRAS gene as one of the founder carcinogenic mutation in the genome [1]. Although it can be mutated at a low percent in almost any cancer types, higher than 10% mutation rates characterizes only ovarian and endometrial cancers (Fig. 1). NRAS is the second most frequently mutated. In malignant melanoma, the mutation rate is around 20%, and a 10% rate characterizes colorectal cancer and hematopoietic malignancies (Fig. 1) [4, 5].

HRAS is rarely mutated in human cancers with a > 10% rate found in only bladder and cervical cancers. There is a discrepancy between the high rates of KRAS mutations in pancreatic cancer by comparing the COSMIC database and individual large cohort reports. The local (homogenous) patient cohort always provides significantly higher mutation frequencies than the COSMIC database, suggesting that there must be geographical differences, even in this high incidence rates (> 80% *versus* 60%, Refs. 4, 5). Usually, these global figures of RAS mutation rates in various cancers are only valid in a given country (typical example is colorectal cancer) [6]. Lung cancer is different. KRAS mutation rate of lung adenocarcinoma is the highest in Europe followed by North America but lowest in India and China (Refs. 7–10, Table 1). Interestingly, such geographical differences can also be found for EGFR mutation rates of lung adenocarcinoma with an opposing trend: highest in China and India and much lower in North America and Europe (Table 1), suggesting different carcinogenic events behind lung adenocarcinoma worldwide. It is also evident that the oncogenic driver mutations are usually mutually exclusive in one particular signaling pathway, in this case in EGFR.

**Table 1:** KRAS/EGFR mutation rates in lung adenocarcinoma in various countries

Mutation	Germany [7]	USA [8]	China [9]	India [10]
KRAS	33%	25%	8%	5%
EGFR	11%	17%	49%	29%

It is of interest that within one cancer type, KRAS mutation may occur in a given specific histotype. In the case of lung cancer, although all induced mostly by smoking, KRAS mutation is specific to adenocarcinoma but absent in small cell lung cancer. If present in squamous carcinoma, then it is a mixture of adeno and squamous forms [11]. On the other hand, in the same histotype of cancer, KRAS mutation frequency can be different by anatomy. In colorectal adenocarcinoma (which is the almost exclusive form), the right-sided cancers are more aggressive than left-sided ones and respond poorer to therapies. In parallel to this biology, the KRAS mutation incidence is significantly more frequent in tumors of the right side as compared with the left [12].

### Mutation patterns of KRAS and their driver function

**Fig. 2.**
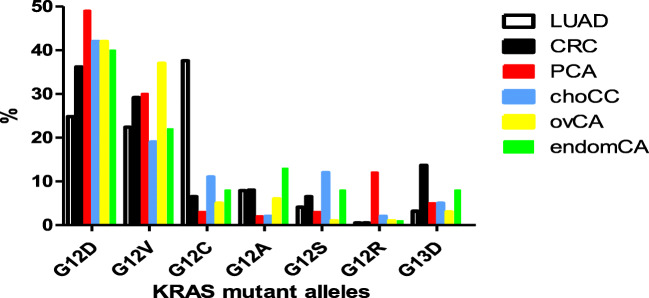
KRAS exon2 mutant allele incidences in various cancers. Data are expressed in %. *LUAD* lung adenocarcinoma, *n* = 579, 2nd Department of Pathology, SU; *CRC* colorectal cancer, *n* = 560, 2nd Department of Pathology, SU; *PCA* pancreatic cancer, *n* = 2661 (Ref. 14); *choCA* cholangial cancer, *n* = 255 (Ref. 13); *ovVA* ovarian cancer, *n* = 410 (Ref. 13); *endomCA* endomatrial cancer, *n* = 306 (Ref. 13).

KRAS mutation pattern is also tumor type specific. We have compared the top six KRAS mutant cancer types for exon2 mutation patterns using our data of routinely tested lung (*n* = 579) and colorectal cancers (*n* = 560) (2nd Department of Pathology, Semmelweis University) and large patient cohorts of cholangiocellular (14, *n* = 255), ovarian (Ref. 13, *n* = 410), and endometrial cancers (Ref. 13, *n* = 306), as well as a large cohort of pancreatic adenocarcinoma (Ref. 14, *n* = 2661). This analysis indicated that G12D followed by G12V mutations are the most frequent across tumor types but with interesting variations. G12D mutation incidence is the highest in pancreatic cancer and the lowest in lung adenocarcinoma. In the case of G12V mutation, ovarian cancer is the most frequent and cholangial cancer of the least. The most frequently cited curiosity of KRAS is the G12C mutation which is by far the most frequent in lung adenocarcinoma (approaching 40%) and significantly less frequent in other tumors (around 10%). The other mutation forms of exon2 are much less prevalent but again tumor type-specific patterns can be found again: G12A mutation characterizes endometrial cancer, G12S is cholangial cancer-specific, and G12R is clearly a pancreatic cancer mutation while G13D is a colorectal cancer mutation, suggesting unique carcinogenic effects (Fig. 2).

These mutational patterns are associated with various carcinogens and/or mutagenic processes such as DNA repair deficiencies and therefore are unique signatures of various cancer types [15]. There are 30 different mutation signatures of human cancers [16]. Data indicated that the lung adenocarcinoma KRAS-G12C mutation is associated with signature4, the smoking-related mutation signature characterized by C > A transitions. Unexpectedly, G12D and G13D mutations of KRAS characterized by C > T transitions are associated with mismatch repair deficiency signatures of stomach and endometrial cancers. It is of note that in experimental lung cancer models, a similar association between G12D mutation of KRAS with genomic instability was observed [17]. On the other hand, analysis of asbestos-induced lung cancers demonstrated that although KRAS mutation rate is significantly high but it is related to the smoking-induced mutations, not the asbestos effects [18]. Irradiation is another etiological factor of lung cancer, but its mutational signature did not show any association with KRAS mutation in irradiation-induced lung cancers [19].

Each cancer is characterized by a well-defined set of mutations, which were initially divided into drivers and passengers [20] but later classified as major or mini drivers [21]. Oncogenic drivers are those mutations, which confer not only transforming capacity but also a selective advantage for those cells in which it occurs. The selective advantage of typical oncogenic mutations can be defined by normalization to its mutational incidence in various cancers. Such an analysis of KRAS mutations in pancreatic cancer identified that the G12R mutation has the highest selective advantage. Analysis of lung adenocarcinoma demonstrated that the most prevalent G12C mutation of KRAS was associated with smoking, and was a major driver, while the G12D and G12V mutations are far less effective and classified as mini drivers. Interestingly, in colorectal cancer, the most prevalent KRAS mutations, G12D and G12V, are weak drivers unlike BRAF600E which is a major driver. In endometrial cancer G12V, G12D, and G12A are all major drivers with strong selective advantages [21]. This information may be important in the future when we are considering the prognostic or predictive power of these various KRAS mutations in various cancer types. From this analysis, it can be concluded that various oncogenic mutations of KRAS may function differently [5, 22, 23] and may have different roles in carcinogenesis.

### Allelic imbalance of KRAS

Oncogenic mutations can be heterozygous or homozygous affecting mutant allele frequency and protein expression. There are relatively few reports on KRAS allelic issues; however a recent report attempts to analyze this important question [24]. Experimental data already suggested that the presence of the wild type-allele of KRAS in the presence of a mutant can have a fundamental effect on the efficacy of the transformation process as well as on the function of the RAS signaling pathway [25]. Homozygous mutation of KRAS can lead to senescence, suggesting that physiologically this “onco”-gene can be considered as a tumor suppressor [26]. Analysis of a large patient cohort, as well as the TCGA database, revealed that allelic imbalance of KRAS is very frequent (55%) in cancers. Heterozygous loss of the wild-type allele is most frequent in lung adenocarcinoma but also very frequent in colorectal or pancreatic cancer (Ref. 24, Table 2). Alternatively, copy number, gain of the mutant allele, is the most frequent in pancreatic cancer and is relatively infrequent in lung adenocarcinoma, and true gene amplification is relatively rare (10–20%). These data suggest that among KRAS mutant cancer types, the mutant gene dose relative to the wild type can be significantly different, as a result of the mechanisms of allelic imbalances, most probably affecting the level of mutant gene expression which can affect protein levels. Allelic imbalances of mutant KRAS according to these data are as frequent as heterozygosity. In all three KRAS cancer types, loss of the wild-type allele is the primary cause of homozygosity which is followed by gains in copy number [24]. These data suggest that there are two types of KRAS mutant human cancer: one containing wild-type allele, which can affect mutant KRAS signaling and, alternatively, where the mutant allele is exclusively expressed (homozygosity) [5, 22, 23].

**Table 2:** Various forms of KRAS allelic imbalances in cancer [24]

	Pancreatic cancer	Colorectal cancer	Lung adenocarcinoma
Heterozygous loss of wild-type allele	47%	67%	74%
Low copy gain of mutant allele	35%	14%	16%
Amplification of mutant allele	18%	19%	10%

### Co-occurring mutations in KRAS mutant cancers and gene expression signatures

#### Lung adenocarcinoma

An analysis of a large patient cohort of KRAS mutant lung adenocarcinoma specifically looked at the co-occurring mutations and found that the most frequent co-mutation in this cohort is TP53 (appr. 40%) followed by STK11, KEAP1, and ATM. Above the 5% mutation levels are SMARCA4, ROS1, and PI3CG [27]. The STK11, KEAP1, and TP53 mutations have been previously observed in KRAS mutant lung adenocarcinomas [28–30]. It is of note that MET and HER2 amplifications are also relatively frequent in KRAS mutant lung adenocarcinomas, while EGFR or BRAF co-occurring mutations are very rare (~1%). It was an unexpected finding that the KRAS mutation type significantly affects co-occurring mutations (i.e., G12C was associated with ERBB4 mutation or HER2 amplification, G12V mutation with PTEN mutation while G12D mutation with PDGFRA mutation) [27].

An analysis of the TCGA’s KRAS mutant lung adenocarcinoma cohort defined a 4-gene signature of mutant tumors (overexpressing FOXRED2, TOP1, PEX3, and ABL2). This was completely different from KRAS-amplified or KRAS wild-type deleted groups [31]. In another study of large public datasets of the KRAS mutant lung adenocarcinoma [32], three subtypes were observed based on gene expression signatures, which were also characterized by different co-mutations, i.e., cluster1/CDKN2A/2B-mutant/LOH, cluster 2/TP53 mutant, and cluster3/LKB1 mutant (Table 3). These clusters did not show any association with KRAS mutation types. Cluster 1 represents a unique subset of lung adenocarcinoma with low TTF1 expression, a characteristic miR signature (miR-31, miR-192, miR-194, miR-215) and mucinous histological features. In cluster 3, mutation of KEAP1 is frequent as well as the inactivation of the LKB1-AMPK pathway. On the contrary, cluster 2 of KRAS mutant lung cancer was characterized by the highest mutation burden, an active immunoediting and high PDL1 expression.

**Table 3:** Subclassification of KRAS mutant lung adenocarcinomas by expression signatures [30]. *m* = mutant

Cluster 1 CDKN2A/2Bm	Cluster 2 TP53m	Cluster 3 LKB1m
VNN1 TFF2 SHC3TC2 ASAP2	SLC16A14 SEC11 ENO3 BMP6	SPRYD5 ICAM1 ARHGAP20

#### Colorectal cancer

A major oncogenic driver in colorectal cancer is KRAS with an average mutation rate of 40%. However, major drivers are oncosuppressors, APC (~70%), and TP53 (~50%) [33]. Co-occurring mutations of KRAS and APC are very frequent (~80%) as well as KRAS and TP53 (~40%). Another oncosuppressor which is frequently mutated in colorectal cancer is PTEN, which also could be co-mutated with KRAS (~25%). On the other hand, co-occurring mutations of KRAS and BRAF are very rare (~1%), and simultaneous mutations of PIK3CA are also relatively rare (~10%). TGFBR signaling pathway alteration (SMAD4) can occur simultaneously with KRAS mutation, although at lower frequency (~20%). It is of note that a Ca-receptor RYR2 is frequently mutated together with KRAS (~35%). It is equally interesting that in KRAS mutant colorectal cancers, simultaneous mutations of RYR2, MUC15, and FAT3 are frequent. On the other hand, mismatch repair deficiency is not associated with KRAS mutation in colorectal cancer [34]

A major success was the identification of consensus molecular subtypes (CMS) in colorectal cancer [35]. CMS1 is characterized by MSI status, BRAF mutation, high immune cell infiltration, and activation but also upregulation of checkpoint inhibitors, PDL1, and CTLA4 [35, 36]. CMS2 displays high chromosomal instability, WNT/MYC pathway activations, and low level of infiltrating immune cells. CMS3 is the prototype of KRAS mutant tumors, while CMS4 has a mesenchymal phenotype characterized by TGF-β activation and high stromal and immune cell infiltration [35, 36]. CMS3 is characterized by metabolic activation including arachidonic acid synthetic pathways. This signature also demonstrates NOTCH signaling activation [35]. Later studies also revealed that in KRAS mutant colorectal cancers, STAT1, CXCL10, and HLA-II are all downregulated [37].

#### Pancreatic cancer

In pancreatic cancer, the predominant oncogenic driver is KRAS mutation, which frequently occurs together with TP53 (80%) and/or CDKN2A (40%) [38]. Homologous recombination repair deficiency is one of the hallmarks of a subset of pancreatic cancer, and mutations of DNA repair genes (BRCA1/2, CHECK2, ATM, PALB2) may occur together with KRAS (20%). Simultaneous mutation of KRAS and BRAF is very rare in pancreatic cancer, similar to mutations of the lipid signaling pathways. On the other hand, mutations of the TGFRB signaling pathway (SMAD2–4 or TGFRB) can frequently occur together with KRAS in this tumor type (~40%). [39]

Gene expression signature of KRAS mutant pancreatic cancer is not defined. A 6-gene prognostic signature was defined recently which composed of CXCL11, FSTL4, SEZ6L, SPRR1B, SSTR2, and TINAG, the high expression of which defines poor prognosis. However, the association of this signature to KRAS mutation has not been evaluated, although KRAS mutation itself is a poor prognostic factor of pancreatic cancer, suggesting a possible link between the oncogenic driver status and this signature [40].

### Intratumoral heterogeneity and clonal fidelity of KRAS mutations during tumor progression

#### Lung cancer

Histologically lung adenocarcinoma is considered one of the most heterogenous cancer types. This heterogeneity is also reflected in the molecular profiles of the primary surgically resected tumors [41]. In an earlier report of the analysis of sixteen KRAS mutant primary tumors, a very high discordance rate was detected between samples of different regions of the primary tumor. In EGFR mutant tumors, wild type to KRAS mutant conversion of the tumor was frequent (30%), while in KRAS mutant tumors, the wild type KRAS subclonality was frequent (30%) [42]. Accordingly, it is expected that such subclonality variations may affect the status of metastases as well. A recent deep-sequencing study on a small cohort of four KRAS mutant patients found complete concordance between the primary tumor and its metastases [43]. However, there are previous studies with larger patient cohorts of metastatic lung adenocarcinomas where concordance was not observed. In a small cohort (11 pts) of bone metastatic lung adenocarcinoma, five cases were KRAS mutant, but unexpectedly only one case was concordant and wild type to mutant and mutant to wild type conversions were observed using a sensitive detection method [44]. In a smaller cohort of metastatic pairs of thirteen KRAS mutant cases, discordance was detected in half of the cases, and wild type to mutant and mutant to wild type conversions both occurred [45]. The largest cohort studied to date was a collection of 33 primary and metastatic pairs of brain metastatic lung adenocarcinoma with 15 cases of KRAS mutants detected by a sensitive PCR methodology. In five cases, the mutant primary changed in metastasis to wild type, while in seven cases KRAS mutant metastases were detected in wild-type primary tumors [46]. These frequent discrepancies can be explained by the clonal heterogeneity of the primary tumors and technical limitations of methods used to detect minute subclones. However, these data strongly argue for the high variability of mutant allele frequencies of KRAS in combination with clonal heterogeneity, resulting in unexpectedly high discordance rates between primary tumors and corresponding metastases.

#### Colorectal cancer

One of the first cancer types where target therapy was introduced (anti-EGFR antibodies) was colorectal cancer. Since there is still no positive predictor, the identification of a strong negative predictor (i.e., RAS mutations) of anti-EGFR therapy was an important step [47]. Testing for RAS mutation became a routine molecular pathology activity in colorectal cancer leading to several studies on KRAS clonality and primary/metastasis comparisons. Heterogeneity of KRAS mutation in the primary tumor was analyzed in a few studies and found to be 10–20% [48, 49]. Interestingly, locoregional lymphatic metastases demonstrated a striking 27% discordance rate [48], a much higher rate than in distant metastases based on analysis of one lymph node metastasis, and this high discordance rate still can be observed by analyzing more than one lymphatic metastases [50]. Visceral metastases were rarely discordant for KRAS mutation status (~10%) [51, 52], but there are studies which discriminated between various metastatic organs and found a much higher discrepancy rate (~30%) in case of lung metastases as compared with other sites [53, 54]. Interestingly, wild type to mutant and mutant to wild-type conversions both occur in metastases [55]. Anti-EGFR antibody therapy is an effective treatment of metastatic colorectal cancer patients in the case of KRAS wild-type tumors, but recurrence/progression occurs relatively frequently. Analysis of ctDNA of anti-EGFR antibody treated, initially, KRAS wild-type patients demonstrated mutant KRAS in 38% of cases, suggesting a clonal selection of mutant cells in the metastatic lesion [56]. Another study analyzed metastatic lesions of EGFR antibody refractory patients and demonstrated frequent emergence of KRAS mutation (55%) with a wide range of mutant allele frequencies (0.04–17.3%), again confirming a clonal selection of KRAS mutant cells during target therapy [57].

#### Pancreatic adenocarcinoma

Unfortunately, the literature is very limited concerning the intratumoral molecular heterogeneity of primary pancreatic cancer. A deep-sequencing analysis of four cases demonstrated no intratumoral heterogeneity of KRAS mutation. Further, analysis of multiple liver, lung, and peritoneal metastases of these cases demonstrated KRAS mutation is present in all samples [58, 59]. However, the case number is very low, and the observed intratumoral molecular heterogeneity and the metastatic colonization pattern of primary region-specificity might suggest caution concerning homogeneity of KRAS mutation in primary and/or metastatic pancreatic cancer.

### Molecular pathologic considerations of KRAS mutation testing

RAS mutations are single-nucleotide variants and thus relatively straightforward to detect. A variety of methods have been established for this task. The more common ones are quantitative real-time PCR which can be done manually or in semi-automated instruments and sequencing technologies ranging from classical di-deoxy-Sanger sequencing to next-generation sequencing (NGS).

Quantitative PCR is a method to detect the abundance of a specific PCR target by monitoring the creation of the PCR product in real time. Once PCR amplification enters exponential phase, a cycle threshold can be determined which is relative to the abundance of the target. Primers are designed flanking the regions of interest (usually one amplicon for G12/13, one each for Q61, K117, and A146). By using fluorescent probes specific to the wild-type and mutated allele, the presence and allele frequency of the mutation can be determined. qPCR is a very sensitive and reliable technology, and there are a multitude of qPCR systems like the Qiagen Therascreen kit commercially available. However, in the context of RAS testing, qPCR has a few significant drawbacks. First, qPCR will only detect the one mutation that is encoded in the molecular probe. All other mutations will not be detected or, in some cases, make the results hard to interpret due to changes in qPCR efficiency. This problem can be overcome by combining probes for multiple mutations into a single PCR tube which in turn will then give a positive result for any of the mutations, but not determine which of the mutations in the mix is present. These general limitations of qPCR can be somewhat mitigated by using multiple fluorophores or probe abundances but still make testing for multiple mutations simultaneously more difficult while using single probe setups, which require more DNA. Nevertheless, qPCR is a reliable and sensitive tool forming the basis of multiple new technologies. Biocartis has designed an instrument which combines DNA extraction, qPCR, and reporting in a single, stylish machine, the “Idylla” system, which has received good reviews in the literature [60, 61]. Another method which even further enhances the sensitivity of qPCR is the digital droplet technology, mainly marketed by Bio-Rad with the QX200 system. In this setup, individual DNA molecules are amplified by qPCR in single oil micelles and then analyzed by a flow-cytometry like approach. This methodology uses minimal DNA, and through the high dilution of the DNA sample PCR inhibitors are removed, resulting in reliable and ultrasensitive results well suited for liquid biopsy analyses [62].

Another main technology to detect RAS mutations is DNA sequencing. Historically, Sanger sequencing has been the method of choice to detect mutations in tumor DNA. Sanger sequencing is still a versatile technology, as it can read long stretches of DNA up to 800 base pairs. It can detect nucleotide variations down to approximately 10% allele frequency and is very sensitive for insertions and deletions. As Sanger sequencing is a seasoned technology, it has been fully automated and consumable costs are low. However, Sanger sequencing still can only analyze a single amplicon in a PCR setup and thus also needs multiples of input DNA for analysis of mutations further apart than the maximum amplicon length.

Pyrosequencing has been introduced to the community in the late 1990s and uses luciferase to generate a light signal when a specific nucleotide is incorporated into a DNA strand. Using pre-amplification and alternating flows of the four nucleotides, the light signal can reveal the sequence of DNA molecules. The technology has been marketed by Qiagen in their PyroMark instruments and was later developed further to give rise to the first NGS platform, the Roche 454 instrument. Pyrosequencing is a more sensitive method for mutation detection than Sanger sequencing, allowing reliable variant calling down to 1% allele frequency. Assays can be purchased commercially as kits for laboratory development. Pyrosequencing is still only able to analyze a single amplicon but presents a middle ground between qPCR and Sanger sequencing, combining high sensitivity with the ability to determine all present variants in a DNA stretch.

The newest technology for RAS mutation testing is next-generation sequencing. Currently there are two ecosystems on the market, namely, Illumina and Ion Torrent. NGS builds upon the initial concepts of pyrosequencing but uses fluorescence markers or pH measurement to determine the sequence of DNA nucleotides. Briefly, the genomic regions of interest are selected and amplified either by multiplex PCR or by hybrid bait capturing. Subsequently, the individual molecules of the DNA library are clonally amplified and immobilized as a DNA ball on glass slides or in wells of a semiconductor device. Finally, universal primers bind to adapter sequences in the DNA balls, and second-strand synthesis is performed either with fluorescent nucleotides followed by optical detection or with unmodified nucleotides followed by detection of incorporation by pH measurement. Both technologies are able to produce millions of reads (short DNA sequences) in the course of hours providing a system, which can sequence large stretches of DNA with very high sensitivity (usually 3–5% allele frequency). The technology has matured over the last 6 years and is now a routine method for mutation analysis in solid and liquid (hematologic) tumors available in many laboratories. The incorporation of molecular barcodes into individual template molecules can further increase sensitivity down to 0.1% allele frequency at the cost of higher sequencing need. This modification makes NGS also applicable to liquid biopsy analysis.

RAS mutation testing is usually restricted to the mutation hotspots so that relatively few regions need to be analyzed. NGS sequencing has relatively high per-sample costs, so it is usually not cost effective to do only RAS testing in a tumor sample. NGS has high multiplex capability so usually NGS panels for colorectal and lung carcinoma or melanoma analyze mutational hotspots in many oncogenes often combined with the full-coding sequence of prominent tumor suppressors like TP53. This panel analysis allows a broader view on the biology of the tumor and has a higher chance of finding druggable targets than RAS testing alone.

Many RAS testing assays can be purchased as IVD-certified tests. This certifies a rigorous validation of the test itself and a very close monitoring of the kit production. IVD-certified tests are usually more expensive but can reduce the risk of technical errors especially in the non-sequencing technologies like qPCR. With the advent of the European legislation on medical devices (especially the IVDR 2017/746), the use of IVD tests is strongly encouraged following FDA/US recommendations and such testing would provide L1 level genetic marker evidences [63, 64]. If DNA is sequenced, the inherent quality control of sequencing data and the direct nature of sequence information makes interpretation of the results more reliable and might allow for the more liberal use of research use only reagents. In general, RAS testing is a robust technique, as it usually only includes well-known hotspot point mutations and thus can be well controlled with positive and negative control samples in qPCR. One possible problem in NGS analysis is barcode bleeding, i.e., the miss-assignment of reads between patients. It can be caused by contamination of adapter molecules used in the library preparation or through sequencing errors in the barcode sequence. Both errors lead to cross-contamination and the presence of mutations prominent in one sample with low frequency in another sample. Another common problem in mutation testing is artifacts introduced due to formalin fixation and prolonged storage of paraffin blocks. These artifacts are mostly G > A and C > T conversions due to deamination of cytosine. In older paraffin blocks, these artifacts can be present at allelic fractions of up to 10% and above which might lead to false-positive results. Deamination artifacts can be addressed by duplicate analyses from the same DNA, as they are stochastic and not likely to be present in both duplicates. Another possibility is the treatment of the DNA with Uracyl-de-Glycosylase (UDG) which removes deaminated Cytosine (Uracil) and thus reduces the abundance of these artifacts. In all cases care must be taken if several C > T and G > A low level mutations are present in a sample as this is very indicative of technical artifacts and a highly degraded sample which needs special care in analysis, Table 4.

**Table 4:** Comparison of common RAS mutation detection systems

Technology	DNA requirement	Sensitivity	Turnaround time	Cost
qPCR	50-300 ng / hotspot	0,1%	1d	25 €
qPCR (automated)	1–3 sections	1%	1d	350 €
ddPCR	5 ng/hotspot	0,01%	1d	25 €
Sanger sequencing	100 ng/amplicon	10%	2d	15 €
Pyrosequencing	100 ng/amplicon	1%	2d	25 €
NGS	10–100 ng/patient	5%	4d	150–450 €
NGS (MBC)	10–100 ng/patient	0.1%	4d	350–650 €

All technologies require an initial investment ranging from 25 (qPCR) to 250 k€ (NGS). DNA extraction time was excluded from TAT calculation. Cost estimates depend on consumables supplier and NGS panel size and should be interpreted with care. *MBC* molecular barcoding

In light of this, it is clear that the interpretation of genetic testing results must be based on the knowledge of the underlying technology, the tumor cell content of the sample as well as the clinical level of evidence of the genetic marker (RAS mutation) [63, 64]. It is important to keep in mind that RAS mutations are driver mutations and will usually be present at an allelic fraction of 50% of the tumor cell content of the sample at least in solid tumors where subclonal events are relatively rare. Reforming a plausibility check of the allelic ratio of a RAS mutation with regard to the tumor cell content of the sample is simple and highly advisable. However, in practice, there are primary or metastatic tumors where mutant KRAS is subclonal, and a low % clone (variant allele frequency) can result in R1 level evidence of resistance [63] to a target therapy. There are currently no guidelines as to defining the threshold levels of mutant RAS to influence therapy. Rather a yes/no rule is applied in the absence of biological or clinical data.

Liquid biopsy (i.e., blood plasma) is a new and emerging analytic target in molecular pathology. It has shown great potential in patients with hard-to-reach tumors or where biopsy is not an option (e.g., in repetitive testing for treatment monitoring) and could potentially also be used as a minimal invasive reflex test. Circulating, cell-free DNA (ccfDNA) extracted from blood plasma is a unique target. On the one hand, in most patients, it contains only a very small fraction of tumor-derived DNA necessitating highly sensitive technologies, currently qPCR, ddPCR, and NGS. On the other hand, as there is no formalin fixation or prolonged storage involved, ccfDNA gives sequencing results with minimal artifacts. By using ddPCR or NGS with molecular barcoding, it is possible to detect very few (1–3) molecules of mutated tumor DNA in a milliliter of blood and identify EGFR, PIK3CA, or RAS hotspot mutations without the need for tissue retrieval by biopsy or operation. As such, liquid biopsy testing is now a common routine method for EGFR resistance mutations in tyrosine kinase inhibitor-treated lung carcinoma and for PIK3CA mutations in breast cancer; however, it is not a routine test for RAS mutation. This is a significant problem when EGFR-targeted therapies fail and resistance/progression of the disease occurs, quite frequently in the form of the emergence of mutant RAS carrying clones [56, 57]. Furthermore, target therapy of the mutant KRAS would require development of appropriate companion diagnostic(s).
